# Influenza Virus PB1-F2 Protein Induces Cell Death through Mitochondrial ANT3 and VDAC1

**DOI:** 10.1371/journal.ppat.0010004

**Published:** 2005-09-30

**Authors:** Dmitriy Zamarin, Adolfo García-Sastre, Xiaoyao Xiao, Rong Wang, Peter Palese

**Affiliations:** 1 Department of Microbiology, Mount Sinai School of Medicine, New York, New York, United States of America; 2 Department of Human Genetics, Mount Sinai School of Medicine, New York, New York, United States of America; Washington University School of Medicine, United States of America

## Abstract

The influenza virus PB1-F2 is an 87-amino acid mitochondrial protein that previously has been shown to induce cell death, although the mechanism of apoptosis induction has remained unclear. In the process of characterizing its mechanism of action we found that the viral PB1-F2 protein sensitizes cells to apoptotic stimuli such as tumor necrosis factor alpha, as demonstrated by increased cleavage of caspase 3 substrates in PB1-F2-expressing cells. Moreover, treatment of purified mouse liver mitochondria with recombinant PB1-F2 protein resulted in cytochrome c release, loss of the mitochondrial membrane potential, and enhancement of tBid-induced mitochondrial permeabilization, suggesting a possible mechanism for the observed cellular sensitization to apoptosis. Using glutathione-S-transferase pulldowns with subsequent mass spectrometric analysis, we identified the mitochondrial interactors of the PB1-F2 protein and showed that the viral protein uniquely interacts with the inner mitochondrial membrane adenine nucleotide translocator 3 and the outer mitochondrial membrane voltage-dependent anion channel 1, both of which are implicated in the mitochondrial permeability transition during apoptosis. Consistent with this interaction, blockers of the permeability transition pore complex (PTPC) inhibited PB1-F2-induced mitochondrial permeabilization. Based on our findings, we propose a model whereby the proapoptotic PB1-F2 protein acts through the mitochondrial PTPC and may play a role in the down-regulation of the host immune response to infection.

## Synopsis

PB1-F2 is a short polypeptide encoded by influenza viruses. While the role of this viral protein is not completely understood, it is known to localize in the mitochodria of the infected cell and to promote cell death. The authors found that PB1-F2 sensitizes cells to death through interactions with two mitochondrial proteins, ANT3 and VDAC1. These interactions promote the permeabilization of the mitochodria and facilitate the release of mitochondrial products that trigger cell death (apoptosis). PB1-F2-mediated cell death through the mitochondria is likely to contribute to the pathogenicity of the influenza virus.

## Introduction

Influenza virus infection results in the activation of cellular pathways aimed at inhibition of viral replication and induction of an antiviral state [1]. To overcome the antiviral signaling, influenza viruses evolved accessory proteins, such as NS1 and PB1-F2, that have been proposed to down-modulate different aspects of the host immune response [2,3]. While the NS1 protein has been shown to play a role in the inhibition of the type I interferon response, the function of the PB1-F2 protein remains elusive.

PB1-F2 is a novel 87-amino acid protein serendipitously identified in an alternate reading frame of the *PB1* gene of the influenza A/PR/8/34 virus [3]. Initial studies revealed that the protein localizes to mitochondria resulting in the alteration of mitochondrial morphology, dissipation of mitochondrial membrane potential, and cell death, which was more pronounced in cells of immune origin [3]. The basic amphipathic helix in the C-terminal region of the PB1-F2 protein was subsequently determined to be responsible for its mitochondrial localization [4,5]. Synthetic peptides derived from the C-terminal domain of the protein were shown to have an ability to oligomerize and nonspecifically permeabilize lipid bilayer membranes [6,7], properties observed with some known cellular mitochondrial apoptotic mediators [8,9]. Despite these findings, however, the precise mechanism and function of PB1-F2-induced apoptosis remains unclear.

Regulation of the mitochondrial permeabilization has been implicated in the life cycle of several known human pathogens [10,11]. Indeed, stable cell lines overexpressing the antiapoptotic proteins of the Bcl-2 family are less permissive to influenza viral replication than their parental cell lines [12–14], highlighting the importance of the role of the mitochondrial apoptotic pathways in influenza virus pathogenesis.

Cellular apoptotic signaling to mitochondria proceeds through activation of the members of the proapoptotic Bcl-2 family BH3-only proteins such as Bid, which exert their effects through induction of the release of several mitochondrial apoptotic mediators, such as cytochrome c, apoptosis-inducing factor, endonuclease G, Smac/Diablo, and Omi/HtrA2 [15]. The exact mechanism leading to the mitochondrial permeabilization is still under investigation, but is known to involve cellular apoptotic mediators of the Bcl-2 family, such as Bak and Bax, and proteins constituting the permeability transition pore complex (PTPC), such as the adenine nucleotide translocator 3 (ANT3) in the inner mitochondrial membrane and the voltage-dependent anion channel 1 (VDAC1) in the outer mitochondrial membrane [16–19].

In view of the possible contribution of the PB1-F2 protein to influenza viral pathogenesis, we sought to determine the role of the protein in modulation of host immune response and to further elucidate its mechanism of action. We show that the mitochondrial permeabilization by the PB1-F2 protein renders cells sensitive to the proapoptotic effect of tumor necrosis factor alpha (TNFα) through tBid signaling. Furthermore, our results indicate that PB1-F2-induced apoptosis proceeds through a unique mechanism involving its interaction with the ANT3 and VDAC1 proteins of the PTPC at the inner and outer mitochondrial membranes, respectively. With the use of ANT3-specific inhibitor, we conclude that PB1-F2 directly permeabilizes mitochondria in an ANT3-dependent manner. The results of our studies provide a deeper insight into the function of the PB1-F2 protein and underscore the role of the PTPC in mitochondrial permeabilization and cell death in influenza virus-infected cells.

## Results

### PB1-F2 Protein Sensitizes Transfected Cells to Apoptotic Stimuli

**Figure 1:**
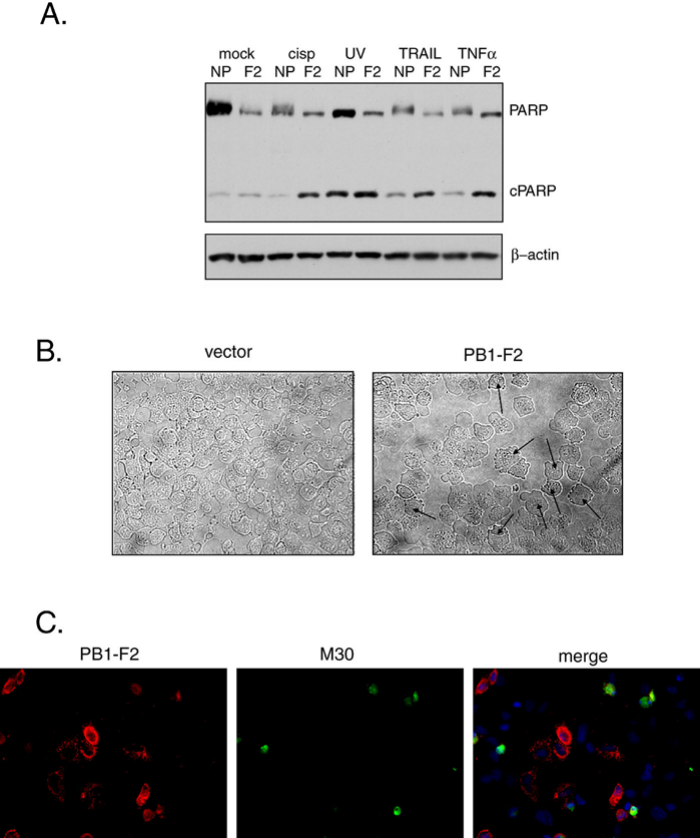
PB1-F2 Protein Sensitizes Cells to Apoptosis in Response to Cytotoxic Stimuli (A) Expression of PB1-F2 protein enhanced cleavage of poly-A ribose polymerase (PARP) in response to cytotoxic agents. 293T cells were transfected with either PB1-F2 (F2) or influenza virus NP (NP) for 24 h and were subsequently treated for 6 h with 50 ng/ml TNFα, 5 ng/ml TNF-related apoptosis-inducing ligand, 100 μM cisplatin, or UV-irradiation at 60 J/m^2^ as indicated. All cells were collected 6 h later, lysed, and processed by immunoblotting for cleaved PARP. (B) PB1-F2 protein predisposes cells to death by anoikis. A549 cells were transfected either with empty vector or with PB1-F2 and trypsinized 24 h post-transfection. Cells were incubated in PBS with 0.3% BSA for 15 min, pipetted onto microscope slides, and changes in cell morphology (membrane blebbing indicated by arrows) were visualized by phase-contrast microscopy [22]. (C) A subset of PB1-F2-expressing cells undergoes apoptosis. A549 cells were transfected with HA-tagged PB1-F2 for 24 h and stained for HA epitope and cleaved cytokeratin (M30).

We investigated whether transient expression of the influenza virus PB1-F2 protein would enhance cell death by intrinsic and extrinsic proapoptotic stimuli (Figure 1). To achieve maximal transient transfection efficiency of the PB1-F2 protein, the proapoptotic effect of the agents was initially assayed in PB1-F2-transfected 293T cells, where apoptosis was detected by cleavage of poly A ribose polymerase (PARP), a direct substrate of caspase 3 (Figure 1A). The unrelated influenza virus protein, nucleoprotein (NP), which does not induce apoptosis, was used as a control. Reduction of full-length PARP with a concomitant increase in cleaved PARP product was seen in PB1-F2-transfected cells in response to DNA damage caused by UV irradiation and cisplatin, when compared to the control (Figure 1A). Treatment of PB1-F2-transfected cells with 50 ng/ml of TNFα or with 5 ng/ml of TNF-related apoptosis-inducing ligand resulted in significant increase in PARP cleavage, as compared to the control (Figure 1A). In view of the findings that PB1-F2 sensitized cells to both extrinsic and intrinsic apoptotic stimuli, we tested whether the PB1-F2-expressing cells are also sensitized to death due to detachment from the growth matrix (anoikis). The activation of anoikis has also been shown to involve proapoptotic members of the BH3 family [20,21]. Indeed, PB1-F2 also sensitized cells to death by anoikis, as demonstrated by rapid membrane blebbing and fragmentation of trypsinized human lung epithelial A549 cells transfected with a construct expressing PB1-F2 (Figure 1B) [22]. To determine whether the PB1-F2-expressing cells were indeed undergoing apoptosis, we labeled PB1-F2-transfected A549 cells with M30 antibody to caspase-cleaved cytokeratin. Interestingly, in the absence of other apoptotic stimuli, only a small proportion of PB1-F2-expressing cells underwent apoptosis (Figure 1C), which could explain the relatively low proapoptotic effect of the PB1-F2 protein observed in the assays above. Overall, these results suggest that the PB1-F2 protein enhances the effect of cellular proapoptotic stimuli.

### Sensitization to Apoptosis by the PB1-F2 Protein Is Inhibited by Bcl-xL

Since PB1-F2 has been shown to localize to mitochondria [3,4], we investigated whether mitochondrial apoptotic mechanisms are involved in PB1-F2-induced apoptotic enhancement. Mitochondrial permeabilization by cellular apoptotic mediators is controlled by the antiapoptotic proteins such as Bcl-xL and Bcl-2. Given the dependence of PB1-F2-induced apoptosis on cellular apoptotic factors, we decided to determine whether PB1-F2-induced apoptotic sensitization can overcome the cytoprotective effect of Bcl-xL. For the purposes of our experiments, we used A549 cells to generate a cell line stably overexpressing Bcl-xL protein (A549-Bcl-xL) and a respective control cell line expressing the gene encoding neomycin resistance. The resulting cell lines both exhibited transient transfection efficiencies of around 60% as witnessed by transient expression of green fluorescent protein (GFP; unpublished data).

**Figure 2:**
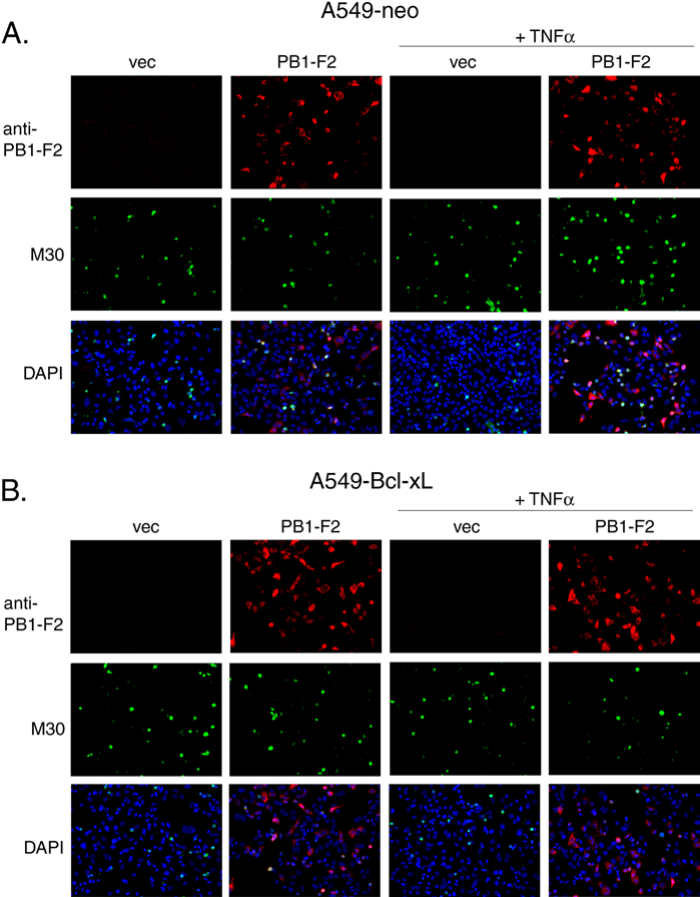
PB1-F2 Protein-Mediated Enhancement of TNFα-Induced Apoptosis Is Inhibited by Bcl-xL A549 cells containing a stably-integrated neomycin resistance gene (A549-neo) (A), or A549 cells stably overexpressing Bcl-xL (A549-Bcl-xL) (B) were transfected with either empty vector or vector encoding HA-tagged PB1-F2. At 24 h post-transfection, cells were treated with 50 ng/ml TNFα, where indicated. Then 6 h post-treatment, the cells were fixed and stained with M30 antibody to cleaved cytokeratin and with anti-HA antibody.

In a control cell line stably transfected with neomycin vector (A549-neo), expression of PB1-F2 enhanced the proapoptotic effect of TNFα, as demonstrated by immunolabeling with M30 antibody specific for cleaved cytokeratin 18 (Figure 2A). Coimmunostaining for PB1-F2 revealed that the PB1-F2-expressing cells were indeed undergoing apoptosis. However, PB1-F2-induced apoptosis in response to TNFα was inhibited in the A549 cell line stably overexpressing Bcl-xL (Figure 2B). Overall these results suggest that the mitochondrial apoptotic pathways are required for PB1-F2-mediated apoptotic enhancement.

### The Influenza Virus PB1-F2 Protein Disrupts Mitochondrial Organization and Induces the Release of Cytochrome C

**Figure 3:**
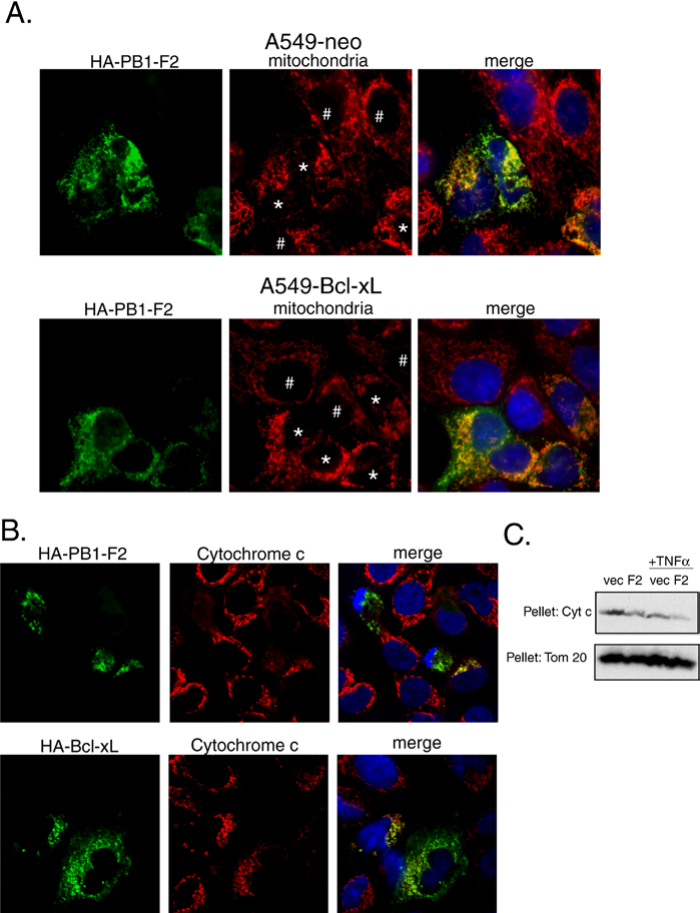
Influenza Virus PB1-F2 Protein Disrupts Mitochondria (A) PB1-F2 disrupts reticulotubular mitochondrial organization. A549-neo and A549-Bcl-xL cells were transfected with HA-tagged PB1-F2 for 24 h and stained with antibody against HA tag and with human anti-mitochondrial serum as a marker for mitochondria. The cells with punctiform mitochondria are indicated by (*), while the cells with normal reticulotubular mitochondrial organization are indicated by (#). (B) PB1-F2 protein induces release of mitochondrial cytochrome c in transfected cells. HeLa cells were transfected with HA-tagged PB1-F2 for 24 h, treated with 50 ng/ml TNFα for 8 h, and stained with anti-HA antibody (green, secondary antibody FITC), anti-cytochrome c antibody (red, secondary antibody Alexa 568), and DAPI (blue). The cells were visualized by confocal microscopy. (C) Subcellular fractionation of 293T cells transfected with PB1-F2. 293T cells were transfected for 24 h and subsequently were either mock-treated or treated with 50 ng/ml TNFα. Cells were subsequently collected and fractionated into the cytosolic and mitochondrial fractions. The amount of cytochrome c remaining in the mitochondrial fraction was detected by Western blot. Tom20 protein was used as a loading control.

To further characterize PB1-F2-mediated apoptotic enhancement, we generated a monoclonal antibody (26D3) specific for the N-terminal domain of the protein, and confirmed by microscopy that the PB1-F2 protein expressed from transfected plasmid localized to mitochondria in A549-neo and A549-Bcl-xL cells (Figure 3A). While in normal cells mitochondria are usually distributed along the microtubular networks, mitochondria of PB1-F2-transfected cells lost the normal tubuloreticular organization and displayed a punctiform appearance (Figure 3A). Interestingly, this effect was not inhibited by Bcl-xL overexpression (Figure 3A), suggesting that disruption of the mitochondrial network is not the primary mechanism for PB1-F2-induced apoptosis.

To further evaluate the effect of PB1-F2 protein on the mitochondrial apoptotic pathways, we analyzed the PB1-F2-expressing cells for release of cytochrome c from the mitochondria. HeLa cells were transfected with hemagglutinin (HA)-tagged PB1-F2, treated with 50 ng/ml TNFα for 8 h, and stained for HA tag and cytochrome c. Expression of the PB1-F2 protein resulted in the release of cytochrome c from mitochondria (Figure 3B) in a subset of cells, as witnessed by diffusion of cytochrome c in the cytoplasm. For a better quantitative measure of the amount of cytochrome c released, 293T cells were transfected with a plasmid expressing PB1-F2 or an empty vector and 24 h later were treated for 8 h with 50 ng/ml TNFα. The cells were subsequently fractionated into cytosolic and mitochondrial fractions, and the amount of cytochrome c remaining in the mitochondrial fraction was determined by Western blot (Figure 3C). Treatment of the PB1-F2 transfected cells with TNFα resulted in enhanced release of cytochrome c from the mitochondria when compared to the vector control.

### PB1-F2 Directly Induces Mitochondrial Permeabilization and Sensitizes Mitochondria to the Proapoptotic Effect of tBid

**Figure 4:**
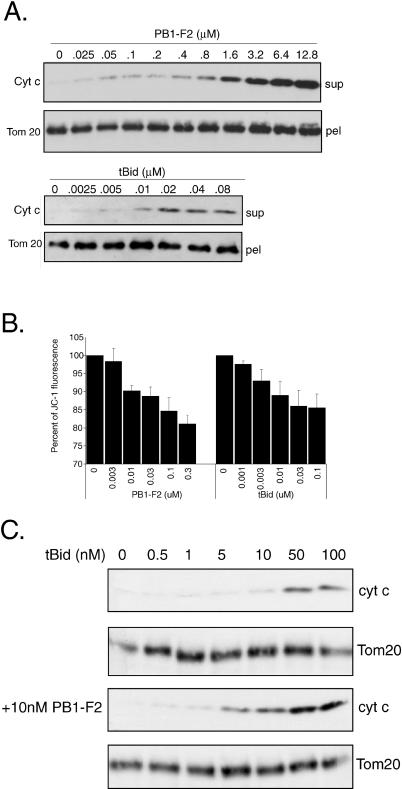
Influenza Virus PB1-F2 Protein Directly Permeabilizes Mitochondria (A) Recombinant PB1-F2 protein caused release of cytochrome c from purified mouse mitochondria. Mouse mitochondria (50 μg total protein) were incubated for 1 h at 30 °C with recombinant protein of interest at indicated concentrations. The amount of cytochrome c released in the supernatant was assayed by immunoblotting using anti-cytochrome c antibody. Recombinant tBid protein was used as a control. The mitochondrial pellet was assayed for the Tom20 protein to ensure an equal amount of mitochondria was used. (B) PB1-F2 and tBid cause dissipation of the mitochondrial membrane potential. Mouse mitochondria (50 μg) were incubated for 10 min at 30 °C with the protein of interest at indicated concentrations. Relative loss in membrane potential was measured by uptake of membrane potential-sensitive JC-1 dye. Data is presented as percent fluorescence relative to mock-treated mitochondria. (C) PB1-F2 protein enhances tBid-induced cytochrome c release. Purified mouse mitochondria (50 μg) were incubated with 10 nM PB1-F2 or mock-treated for 15 min in 20 μl and were subsequently treated with recombinant tBid in the indicated concentrations for 1 h in a total volume of 30 μl. The supernatants were assayed for cytochrome c release by Western blot. The mitochondrial pellet was processed for Tom20 to ensure equal amount of mitochondria used.

To further investigate whether the effect of PB1-F2 protein on the mitochondria is direct, we incubated purified mouse liver mitochondria with recombinant PB1-F2 protein. Recombinant tBid protein was used as a control. Treatment of mitochondria with the PB1-F2 protein resulted in the release of cytochrome c, which increased at higher concentrations, suggesting a direct role for PB1-F2 in promoting mitochondrial outer membrane permeabilization (Figure 4A). This effect was not observed when a recombinant glutathione-S-transferase (GST) protein was used in this assay at similar concentrations (unpublished data). In view of the previous report that the C-terminal region of PB1-F2 protein targets it to the inner mitochondrial membrane, we proceeded to investigate whether PB1-F2-induced mitochondrial permeabilization also involves the inner mitochondrial membrane. For this purpose we used the mitochondrial potential-sensitive dye JC-1 to determine whether incubation of purified mitochondria with recombinant PB1-F2 protein would result in dissipation of the mitochondrial inner membrane potential. The uptake of the JC-1 dye by intact mitochondria results in increased red-orange fluorescence at 590 nm upon excitation at 490 nm. As can be seen from Figure 4B, incubation of the purified mitochondria for 10 min with increasing doses of PB1-F2 resulted in reduction of JC-1 fluorescence at 590 nm, suggesting that the PB1-F2 protein-induced mitochondrial permeabilization involves the inner mitochondrial membrane. Interestingly, while recombinant PB1-F2 protein was, at equimolar concentrations, less effective than tBid in releasing cytochrome c (Figure 4A), it was comparable to tBid in permeabilization of the inner mitochondrial membrane. This suggests that while loss in the mitochondrial membrane potential might not be required for tBid-induced cytochrome c release [23], it may be necessary for PB1-F2-induced mitochondrial permeabilization. These results confirmed previous findings that showed that expression of PB1-F2 protein in cells can lead to dissipation of the mitochondrial membrane potential [3]. Interestingly, as we and others showed, expression of the PB1-F2 protein itself does not cause significant levels of apoptosis [3]. This suggests that the inner membrane permeabilization by the PB1-F2 protein might not be the primary mechanism of apoptosis induction. Rather, we hypothesize that PB1-F2-induced inner membrane permeabilization acting in conjunction with another apoptotic mechanism may be responsible for PB1-F2-mediated apoptotic enhancement illustrated in Figures 1 and 2.

In view of the fact that both intrinsic and extrinsic cellular apoptotic stimuli converge on the mitochondria through signaling of the Bcl-2 family BH3 proteins, we investigated whether the presence of PB1-F2 in suboptimal concentrations would enhance the release of cytochrome c from purified mitochondria by recombinant tBid. Incubation of the purified mitochondria with recombinant tBid results in release of cytochrome c at tBid concentrations as low as 10 nM (Figure 4C). Preincubation of the purified mitochondria with 10 nM PB1-F2 augments the tBid-induced cytochrome c release by a factor of almost ten (Figure 4C). As the PB1-F2 protein was used at a concentration at which it fails to release cytochrome c by itself, the result suggests that the effect of PB1-F2 and tBid is not simply additive. We speculate that PB1-F2-induced permeabilization of the inner mitochondrial membrane may augment cytochrome c release in response to tBid. Overall, these results suggest that sensitization of cells to apoptosis by the PB1-F2 may proceed through its potentiation of the effects of the cellular BH3 family proteins.

Previous studies revealed that the PB1-F2 protein localizes to both inner and outer mitochondrial membranes [3–5]. As the cellular apoptotic proteins were previously shown to act through the mitochondrial proteins controlling permeabilization of the inner as well as outer mitochondrial membranes, we speculate that the observed PB1-F2-induced apoptotic sensitization could proceed via components in both membranes. To investigate possible involvement of mitochondrial proteins in PB1-F2-induced apoptosis, we sought to identify potential mitochondrial interactors of the PB1-F2 protein.

### The PB1-F2 Protein Interacts with ANT3 and VDAC1 Proteins of the Mitochondrial PTPC

**Figure 5:**
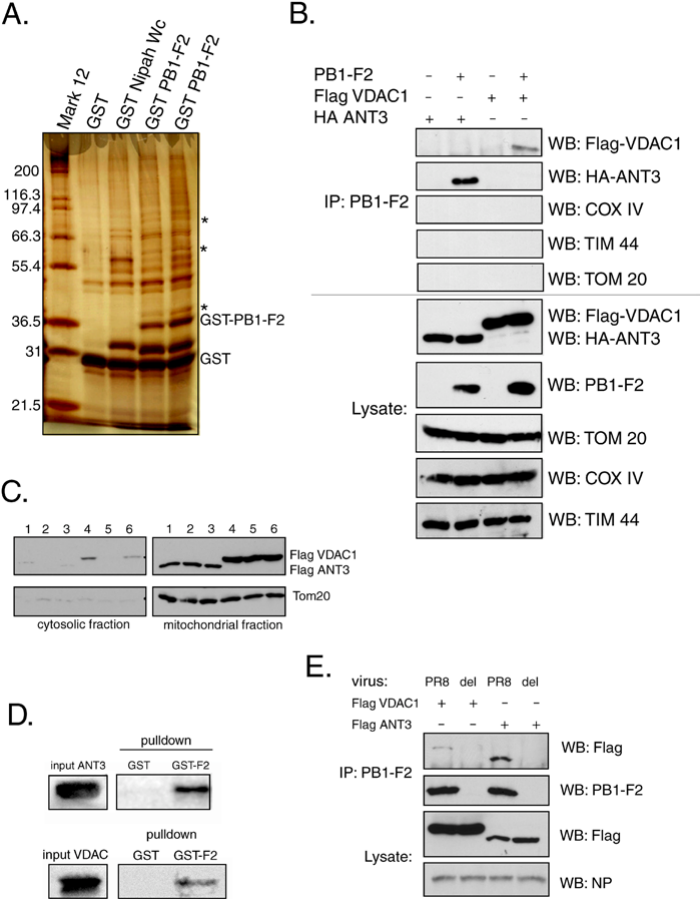
PB1-F2 Protein Interacts with ANT3 and VDAC1 (A) Cellular proteins pulled down with GST-PB1-F2 were separated by 12% SDS-PAGE and silver-stained. The asterisks mark the protein bands that are unique to the GST-PB1-F2 lanes (~36 kDa, 55 kDa, and 80 kDa). Lanes 4 and 5 represent the results of two separate pulldown experiments. (B) PB1-F2 specifically interacts with ANT3 and VDAC1 but not other outer and inner mitochondrial membrane proteins. Interaction of PB1-F2 with ANT3 and VDAC1 was confirmed in 293T cells by coimmunoprecipitation with transfected HA-tagged ANT3 and flag-tagged VDAC1 (top five images). Tom20, COXIV, and TIM 44 proteins were used as the mitochondrial coimmunoprecipitation controls. (C) Transfected ANT3 and VDAC1 localize to mitochondria. 293T cells were transfected with Flag-tagged ANT3 and VDAC1 for 24 h and were subsequently fractionated to generate the cytosolic and mitochondrial fractions. Tom20 protein served as a control for the mitochondrial fraction. Each sample was processed in triplicate (lanes 1, 2, and 3, ANT3; lanes 4, 5, and 6, VDAC1). (D) PB1-F2 directly interacts with ANT3 and VDAC1. ^35^S-labeled ANT3 and VDAC1 were expressed in vitro using a rabbit reticulocyte lysate system and subjected to pulldown with either 5 μg GST (left lane) or GST-PB1-F2 (right lane). Proteins were separated by 12% SDS-PAGE and visualized by autoradiography. (E) PB1-F2 expressed during viral infection interacts with ANT3 and VDAC1. 293T cells were transfected with Flag-tagged ANT3 and VDAC1 proteins and 24 h later were infected with wild-type PR8 virus (PR8) or the virus knocked out for PB1-F2 expression (del) at an MOI of 2. 15 h after infection, the cells were collected and the immunoprecipitation with anti-PB1-F2 polyclonal serum was performed. Influenza virus NP was used as a control to show equal levels of infection between the samples. WB, Western blot.

PB1-F2 protein was expressed in 293T cells as an N-terminal GST fusion protein, and the complexes between GST-PB1-F2 and its interacting proteins were pulled down with glutathione-Sepharose beads. Proteins were subsequently eluted from the beads, separated by SDS gel electrophoresis, and visualized by silver stain. An unrelated polypeptide of similar length fused to GST (GST-Nipah-Wc, containing the last 43 amino acids of the W protein of the Nipah virus fused to GST) was used as a control. Unique protein bands at approximately 36, 55, and 80 kDa specific for the PB1-F2 fusion protein but not GST or the control protein were detected (Figure 5A, lanes 4 and 5). Mass spectrometry analysis of the proteins identified the 36-kDa band as the mitochondrial ANT3, the 55-kDa band as beta tubulin, and the 80-kDa band as cytokeratin. The latter was likely a contaminant of the protein preparation. While interaction of PB1-F2 with tubulin may be important for PB1-F2-induced mitochondrial disorganization (see Figure 3A), we chose to focus on the ANT3 protein, which is known to be involved in mitochondrial apoptosis. Coimmunoprecipitation experiments with transfected HA-tagged ANT3 confirmed the interaction between PB1-F2 and ANT3 (Figure 5B). As expected, transfected HA-tagged ANT3 localized to the mitochodria (Figure 5C).

To verify that the interaction between PB1-F2 and ANT3 was direct, we expressed ^35^S-labeled ANT3 in vitro and performed GST pulldowns on the labeled protein with either GST or GST-PB1-F2 proteins. Only GST-PB1-F2 was able to coprecipitate the in vitro-translated ANT3 protein, suggesting that direct interaction between PB1-F2 and ANT3 is likely (Figure 5D).

Previous studies revealed that the predicted basic amphipathic helix formed by amino acids 69–82 is sufficient for targeting PB1-F2 to the inner mitochondrial membrane [4]. However, immunoelectron microscopy studies had also previously shown that the full-length PB1-F2 protein localized to both inner and outer mitochondrial membranes [3], suggesting that the N terminus of the protein may play a role in targeting the protein to the outer mitochondrial membrane. Thus, while ANT3 was the only component of the PTPC identified by mass spectrometry to interact with PB1-F2, we could not exclude the possibility that PB1-F2 exerts its effect in conjunction with other proteins of the pore complex. Since ANT3 is exclusively an inner membrane protein, we speculated that the outer membrane PB1-F2 may be acting through an additional mechanism. Indeed, immunoprecipitation experiments revealed that PB1-F2 protein also interacted with VDAC1 (Figure 5B), and GST pulldown experiments with in vitro-translated VDAC1 confirmed that the interaction is likely direct (Figure 5D). These findings were surprising, since VDAC1 protein was not identified in the original experiment by mass spectrometry. While it is likely that the VDAC1 protein was missed in our analysis, it is also possible that the N-terminal GST tag may have prevented the interaction of the PB1-F2 protein with VDAC1 within the cell.

ANT3 and VDAC1 appear to be critical components of the pore complex [19]. ANT3 is an inner mitochondrial membrane protein that functions as an antiporter catalyzing the exchange of ATP for ADP [19,24]. In the presence of apoptotic stimuli, ANT3 is believed to undergo major conformational changes resulting in the formation of nonspecific pores in the inner mitochondrial membrane. Apoptotic stimuli also trigger conformational changes in the outer membrane protein VDAC1, which forms pores in the outer mitochondrial membrane. ANT3 and VDAC1 are believed to interact, forming the PTPC, which leads to dissipation of the inner mitochondrial membrane potential and the release of apoptotic mediators from the mitochondrial intermembrane space [24–26].

To eliminate the possibility of nonspecific interaction of PB1-F2 with mitochondrial proteins, we performed PB1-F2 coimmunoprecipitation experiments with the outer mitochondrial membrane transport protein Tom20, the inner mitochondrial membrane protein COXIV, and the protein Tim44, which localizes to the matrix side of the inner mitochondrial membrane (Figure 5B). PB1-F2 protein failed to coimmunoprecipitate with any of these proteins, further confirming the specificity of its interaction with ANT3 and VDAC1 (Figure 5B).

To confirm that the transfected tagged ANT3 and VDAC1 proteins were properly targeted to the mitochondria, we performed subcellular fractionation and determined that the majority of the expressed ANT3 and VDAC1 are indeed present within the mitochondrial fraction (Figure 5C). Furthermore, to determine whether PB1-F2 interacts with ANT3 and VDAC1 within the context of viral infection, we infected Flag-ANT3- and Flag-VDAC1-transfected 293T cells with either a wild-type PR8 virus or with its correspondent virus knocked out for PB1-F2 protein expression. Immunoprecipitation experiments with anti-PB1-F2 polyclonal serum revealed that the PB1-F2 protein interacts with ANT3 and VDAC1 during the viral infection (Figure 5E).

Due to lack of availability of good antibodies specific for ANT3 and VDAC1, we were unable to demonstrate the interaction of PB1-F2 with the endogenous proteins, apart from the interaction shown by mass spectrometry. Thus, to further confirm the specificity of the interaction, we proceeded to identify the interaction domains within the PB1-F2 protein.

### The C-Terminal Domain of PB1-F2 Protein Is Responsible for the Interaction with ANT3, While Both C- and N-Terminal Domains Interact with VDAC1

**Figure 6:**
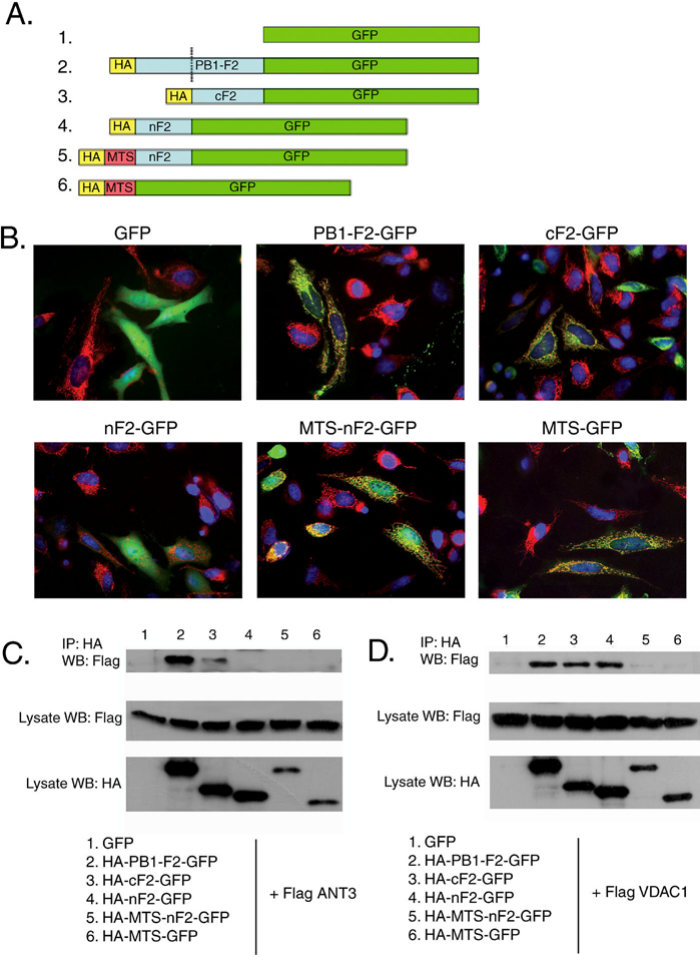
Identification of PB1-F2 Protein Domains Responsible for Interaction with ANT3 and VDAC1 (A) PB1-F2 N- and C-terminal domains were cloned separately as N-terminal HA- and C-terminal GFP fusion proteins as follows. (1) GFP control; (2) full-length fusion protein (HA-PB1-F2-GFP); (3) C-terminal 38–87-amino acid domain fusion protein (HA-cF2-GFP); (4) N-terminal 1–37 amino acid domain fusion protein (HA-nF2-GFP); (5) N-terminal domain fusion protein with cytochrome oxidase mitochondrial targeting sequence (HA-MTS-nF2-GFP); and (6) control MTS-GFP fusion protein (HA-MTS-GFP). (B) Localization of fusion proteins was determined in transfected HeLa cells (green, GFP-fusion protein; red, Mitotracker dye staining for mitochondria; blue, DAPI nuclear stain). (C and D) Interaction of fusion proteins with ANT3 and VDAC1 was determined by cotransfecting 293T cells with each fusion construct and a vector encoding Flag-tagged ANT3 or VDAC1, respectively. Immunoprecipitation was performed with an anti-HA antibody, with subsequent immunoblotting for Flag-tagged ANT3 or VDAC1.

Expression of HA-tagged N- or C-terminal domains of PB1-F2 was unsuccessful and resulted in fragments which appeared to be unstable (unpublished data). To stabilize each part of the protein, we generated HA-tagged GFP-fusion protein constructs of each domain (HA-nF2-GFP and HA-cF2-GFP; Figure 6A). The N-terminal region (HA-nF2-GFP) included amino acids 1–38, while the C-terminal fusion protein (HA-cF2-GFP) included amino acids 39–87 (Figure 6A). In transfected HeLa cells, full-length HA-PB1-F2-GFP fusion protein and HA-cF2-GFP protein localized to the mitochondria, while the HA-nF2-GFP fusion protein was diffusely distributed in the cytoplasm and the nucleus (Figure 6B). Since possible interaction of the N terminus of the protein with its cellular target could be influenced by its subcellular localization, we generated an additional construct fusing the N terminus to the mitochondrial targeting signal of the cytochrome oxidase, which targets the protein to the inner mitochondrial membrane (HA-MTS-nF2-GFP) [27]. The resultant fusion protein localized to mitochondria (Figure 6B). We next determined whether the fusion proteins interacted with Flag-tagged ANT3 and VDAC1 in transfected 293T cells. Both HA-PB1-F2-GFP and HA-cF2-GFP fusion proteins proved to interact with both VDAC1 and ANT3, while the HA-nF2-GFP interacted only with VDAC1 (Figure 6C and 6D). HA-MTS-nF2-GFP protein possessing the inner mitochondrial membrane targeting sequence still failed to interact with ANT3, confirming that the N terminus of the protein is not responsible for the interaction (Figure 6C). Interestingly, the interaction of HA-MTS-nF2-GFP protein with VDAC1 was also greatly reduced when compared to the HA-nF2-GFP protein lacking the MTS (Figure 6D). It is possible that forced localization of the nF2 to the inner mitochondrial membrane may have prevented its interaction with VDAC1 in the outer membrane.

### The PB1-F2 Protein-Mediated Mitochondrial Permeabilization Is Attenuated by ANT3 Blockers

**Figure 7:**
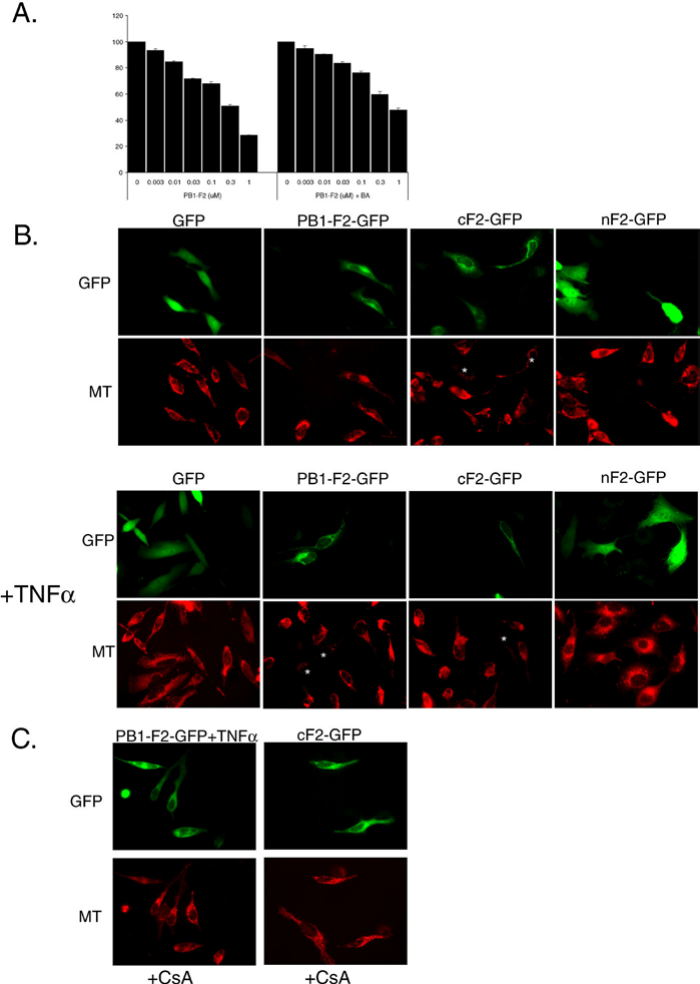
PB1-F2 Protein Induces Mitochondrial Permeabilization in ANT3-Dependent Manner (A) Recombinant PB1-F2 protein was incubated with 50 μg of purified mouse liver mitochondria in the presence or absence of 50 μM BA for 30 min. The mitochondria were further processed for assessment of membrane potential by JC-1 fluorescence at 590 nm. (B) PB1-F2 induces loss of mitochondrial membrane potential in transfected cells. HeLa cells were transfected with GFP fusion constructs of PB1-F2, and 12 h later were treated with 50 ng/ml TNFα for 8 h, where indicated. Cells were subsequently stained with Mitotracker CMXRos Red dye. Cells with dissipated membrane potential are indicated by (*). (C) The mitochondrial permeability transition inhibitor CsA inhibits PB1-F2-induced loss of mitochondrial membrane potential. HeLa cells in presence of CsA were transfected and treated as in (B) and stained with Mitotracker dye.

We further investigated the role of the PTPC in PB1-F2-induced mitochondrial permeabilization. We turned to the known pore complex inhibitors: bongkrekic acid (BA), which was shown to inhibit ANT3 [28], and cyclosporine A (CsA), which binds to the mitochondrial matrix cyclophilin D and also inhibits ANT3. Purified mouse liver mitochondria were treated for 30 min with recombinant PB1-F2 protein in the presence or absence of BA (Figure 7A). Incubation of mitochondria with increasing doses of the PB1-F2 protein resulted in dissipation of the mitochondrial membrane potential as measured by JC-1 fluorescence. This effect was attenuated, although not completely inhibited, when the mitochondria were preincubated with 50 μM BA (Figure 7A).

To confirm the involvement of ANT3 in PB1-F2-induced dissipation of the mitochondrial membrane potential in cells, and to identify the domain of the protein responsible for this permeabilization, we transfected HeLa cells with the PB1-F2-GFP fusion constructs described in Figure 6 and stained the cells with the mitochondrial membrane potential-sensitive Mitotracker CMXRos Red dye. GFP protein was used as a control. Consistent with previous reports [4,5], we found that in the absence of other stimuli, the C-terminal domain of the protein was more effective in dissipating the inner membrane potential than the full-length protein (Figure 7B, upper photomicrographs), as revealed by decreased Mitotracker Red staining in these cells. This is further consistent with our findings that the C-terminal domain of the PB1-F2 protein is responsible for its interaction with ANT3 (Figure 6). Treatment of the HeLa cells expressing the PB1-F2-GFP fusion construct with TNFα resulted in dissipation of the membrane potential, further confirming that the full-length protein requires additional apoptotic stimuli for its effect (Figure 7B). To confirm that the PB1-F2-induced permeability transition indeed proceeds in ANT3-dependent manner, we performed the same experiment in the presence of CsA, a known ANT3 inhibitor (Figure 7C). Treatment of HeLa cells with CsA resulted in preservation of the mitochondrial membrane potential in the cells expressing the cF2-GFP and in the TNFα-treated cells expressing the PB1-F2-GFP protein.

### PB1-F2-Induced Apoptosis Proceeds in ANT3-Dependent Manner

**Figure 8:**
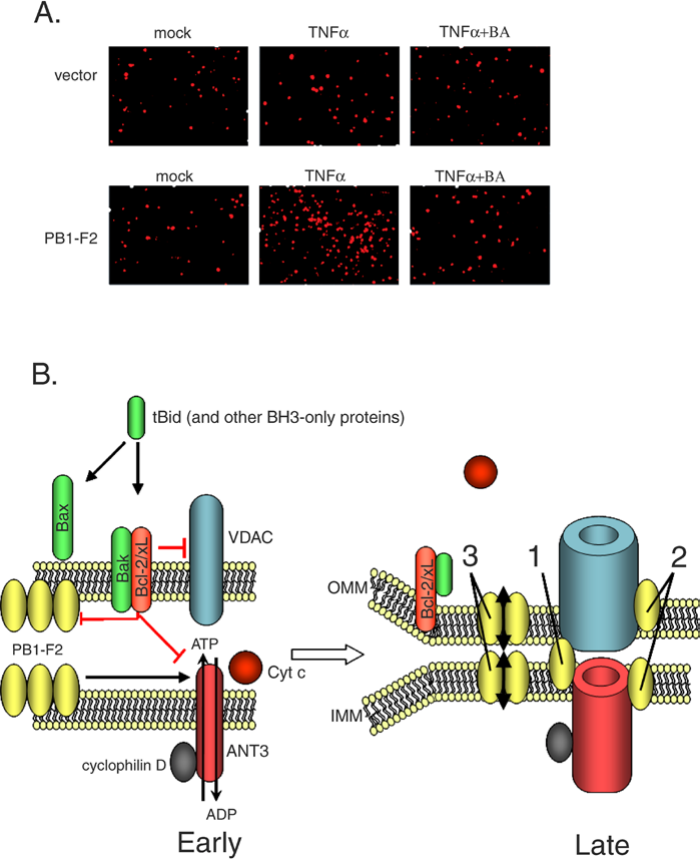
PB1-F2 Induces Apoptosis Acting through Components of the PTPC (A) ANT3 blocker BA inhibits PB1-F2-mediated sensitization of cells to apoptosis. A549 cells were transfected with PB1-F2 for 20 h and then treated with TNFα as indicated, either in the presence or absence of 50 μM BA. The cells were collected 8 h later and stained with M30 antibody to cleaved cytokeratin. (B) Proposed models of PB1-F2 action during infection. During early stages of the infection, PB1-F2 localizes to mitochondria, where it interacts with ANT3 and VDAC and predisposes the mitochondria to permeability transition. Later in the infection, when more PB1-F2 is synthesized, and upon induction of antiviral apoptotic signaling pathways, the mitochondria undergo the permeability transition, which results in the induction of apoptosis. PB1-F2-induced mitochondrial permeabilization can proceed through three possible mechanisms, as indicated on the right graphic: (1) enhancement of the pore complex formation through direct interaction with ANT3 and VDAC1; (2) independent permeabilization of the inner and outer mitochondrial membranes with the help of ANT3 and VDAC1, respectively; and (3) direct permeabilization of the mitochondrial membranes.

To confirm that the PB1-F2-mediated sensitization to apoptosis proceeds in ANT3-dependent manner, we investigated whether BA, an ANT3 blocker, could inhibit this effect. A549 cells were transfected with PB1-F2 for 24 h and were subsequently treated with TNFα either in the presence or absence of 50 μM BA. As can be seen from Figure 8A, BA inhibited TNFα-induced apoptosis in these cells, as compared to the untreated control. Overall, these results, along with the interaction studies, suggest that the PTPC plays a role in PB1-F2-induced mitochondrial permeabilization.

## Discussion

Mitochondrial control of apoptosis is a critical gateway for many cellular apoptotic pathways, whereby mitochondrial permeabilization and release of mediator proteins represent the point of no return in the execution of apoptotic cascades [29]. Permeabilization of mitochondria is thought to occur through two proposed mechanisms, proceeding in either permeability transition-dependent or -independent manner [16,17]. The mechanisms are not mutually exclusive, and it is generally believed that both play a role in the mitochondrial induction of apoptosis, whereby the contribution of each mechanism is dependent upon the apoptotic stimulus and/or perhaps differential tissue-specific regulation [17].

Several viral and bacterial proteins have been shown to induce or inhibit apoptosis through a direct effect on different mitochondrial components [10,11]. Viruses of the herpesvirus family evolved to regulate apoptosis by different mechanisms, with some members encoding Bcl-2 family homologs [30]. Porin proteins of *Neisseria* and the hepatitis B virus X protein interact with VDAC [31–33]. Cytomegalovirus vMIA protein and HIV Vpr proteins affect the permeability transition by targeting ANT3 [34,35], and the M11 protein of myxoma virus targets the peripheral benzodiazepine receptor, another component of the pore complex [36]. Still other viral proteins exert their effect on the mitochondria through yet unidentified mechanisms [10,11].

Knowing the role of influenza virus PB1-F2 protein in apoptosis, we decided to elucidate its mechanism of action and the role that the protein may play in viral infection. The sensitivity to apoptosis of PB1-F2-expressing cells was greatly enhanced in response to different cellular apoptotic stimuli, such as DNA damage, anoikis, and signaling through death receptors (see Figure 1). Similar findings were previously reported for the hepatitis B virus X protein [37]. Since proteins of the BH3 family such as Bid have been implicated in both intrinsic and extrinsic apoptotic signaling [20,22,38], we hypothesized that PB1-F2 may sensitize mitochondria to proapoptotic effects of Bid. Indeed, treatment of purified mitochondria with recombinant tBid in the presence of PB1-F2 resulted in enhanced cytochrome c release (see Figure 4). Furthermore, the proapoptotic effect of PB1-F2 on cells in response to TNFα was blocked by Bcl-xL (see Figure 2), which could occur through inhibition by Bcl-xL of either outer or inner mitochondrial membrane permeabilization. Interestingly, Bcl-xL overexpression did not inhibit PB1-F2-induced disorganization of mitochondria (see Figure 3A). This suggests that alteration of mitochondrial organization by PB1-F2 may proceed through a different mechanism, possibly through its interaction with tubulin (see Figure 5A).

Due to the unstable nature of the PB1-F2 protein and its low expression levels within the cells, the studies described above were performed in cell lines with good transfection efficiencies, such as A549 and 293T cells. The major drawback of this strategy is the transformed nature of these cells, which may alter their apoptotic responses. In particular, as seen from Figures 1 and 2, these cells are normally resistant to proapoptotic effects of TNFα, suggesting that the survival/proinflammatory pathways activated by TNFα exert a dominant effect. Nevertheless, expression of the PB1-F2 protein shifted this balance toward the proapoptotic pathways in both cell lines.

We further evaluated the mechanism of PB1-F2-induced mitochondrial permeabilization and determined that the PB1-F2 protein directly induced cytochrome c release and loss of the mitochondrial inner membrane potential in purified mouse liver mitochondria (see Figure 4A and 4B), suggesting that PB1-F2 may be acting in permeability transition-dependent manner. This speculation was further supported when proteins of the PTPC ANT3 and VDAC1 were identified in our PB1-F2 interaction screen.

To confirm that PB1-F2-induced mitochondrial permeabilization proceeds through the pore complex in an ANT3-dependent manner, we used two known ANT3 blockers, BA and CsA. These inhibitors attenuated the PB1-F2-induced loss of the mitochondrial membrane potential both in purified mitochondria (Figure 7A), and in live cells (Figure 7B). Furthermore, BA inhibited PB1-F2-mediated sensitization of cells to apoptosis by TNFα (Figure 8A). This suggests that, while similarly to the proteins of the Bcl-2 family, the PB1-F2 protein may permeabilize membranes nonspecifically [6,8,9], it induces a permeability transition in the inner mitochondrial membrane in an ANT3-dependent manner.

ANT3 is localized to the inner mitochondrial membrane and contributes to apoptosis induction by forming nonspecific pores that result in dissipation of the inner mitochondrial membrane potential. Direct interaction of ANT3 with several cellular and viral proteins (including the HIV Vpr protein) has been shown to result in mitochondrial permeabilization, leading to apoptosis [10,11]. The idea of the importance of ANT3 involvement in apoptosis has recently been challenged by the finding that the knockout of the mouse homologs of ANT3 did not alter the induction of apoptosis [39]. The results of that work nevertheless suggested that ANT3 does have an essential role in regulating permeability transition by modulating the sensitivity of the pore complex to Ca^2+^ activation and ANT ligands [39]. Similarly to the HIV Vpr, the influenza virus PB1-F2 protein may act as a direct ligand to ANT3 [40].

Interaction of the PB1-F2 protein with both ANT3 and VDAC1 was a surprising finding, since to our knowledge this is the first viral protein shown to interact with components of the pore complex located in both inner and outer mitochondrial membranes. We further determined that, while interaction of PB1-F2 protein with ANT3 occurs through its C-terminal domain (see Figure 6), interaction of PB1-F2 protein with VDAC1 is mediated through both N- and C-terminal regions. Overall, these data suggest a possible complex formation between VDAC1, ANT3, and PB1-F2, whereby PB1-F2 may bridge VDAC1 and ANT3, thus promoting formation of the PTPC (see Figure 8B). Further experiments will be needed to determine whether such a complex is indeed formed within the cell. In this study, we did not further identify the PB1-F2 protein residues responsible for the interaction with ANT3 and VDAC1. In a previous study, the inner mitochondrial membrane targeting signal was mapped to the amphipathic helix within the C-terminal region of the protein, consistent with our finding that the PB1-F2 C-terminal region interacts with the inner membrane ANT3 [4]. The amphipathic helix by itself was sufficient to dissipate the mitochondrial membrane potential, and mutations within the amphipathic helix abolished the mitochondrial localization of the protein [4]. At this point, however, we are unable to separate the mitochondrial-targeting and the ANT3-binding domains of the PB1-F2 protein, since lack of mitochondrial targeting also abolished PB1-F2-ANT3 interaction. In support of our findings, the N-terminal region of the PB1-F2 protein targeted to mitochondria with a heterologous inner mitochondrial membrane targeting sequence failed to interact with ANT3, confirming the specificity of the ANT3 interaction with the PB1-F2 C-terminus.

The induction of permeability transition by PB1-F2 could proceed through three possible mechanisms (Figure 8B). Through its interaction with both ANT3 and VDAC1, PB1-F2 could potentially act as a bridge between the two proteins, enhancing the formation of the pore complex (mechanism 1). Alternatively, the protein could act separately at the inner and outer mitochondrial membranes, in conjunction with ANT3 and VDAC1, respectively (mechanism 2). The latter model is supported by the evidence that PB1-F2 has been shown to localize to both inner and outer mitochondrial membranes and to directly induce membrane permeabilization [3,6]. Recent studies also indicate that, similar to the members of the Bcl-2 family, the PB1-F2 protein is capable of forming multimeric complexes, which are probably responsible for its membrane-permeabilizing activity [7]. Thus, direct permeabilization of the mitochondrial membrane by PB1-F2 protein complexes could account for the third possible mechanism.

We chose to focus our studies on the VDAC1 and ANT3 proteins primarily because these proteins were identified in our interaction screens, while other proteins localized to the outer and inner mitochondrial membranes and the mitochondrial matrix (Tom20, COXIV, and Tim44) were excluded. In addition, these protein isoforms are specifically known to be involved in the mitochondrial permeability transition. We cannot, however, exclude the possibility that the PB1-F2 protein may also interact with other isoforms of ANT and VDAC. In particular, a recent study showed that the VDAC2 isoform is implicated in suppression of mitochondrial apoptosis [41]. The fact that the ANT3-specific inhibitor BA did not completely inhibit PB1-F2-induced inner membrane permeabilization suggests that additional players may be involved (Figure 7A). In addition, while the results of our work propose the role of ANT3 in PB1-F2-induced permeabilization of the inner mitochondrial membrane, further experiments are needed to determine the role of the VDAC1 protein and the function of the PB1-F2 protein in the outer membrane. Our studies are currently limited by the lack of availability of specific inhibitors against the VDAC1 protein. Experiments are currently in progress to individually characterize the role of VDAC1 in PB1-F2-induced mitochondrial permeabilization.

Since the PB1-F2 protein is relatively short-lived and is expressed mainly during the later stages of the infection cycle [3], its proapoptotic effect most likely is not inhibitory to viral replication. Based on our findings, we propose the following model for the mode of PB1-F2 protein action within the cell (Figure 8). Early in the infection cycle, PB1-F2 localizes to the mitochondria, but does not induce a permeability transition due to its low levels present within the cell. This allows for maintenance of cell viability, which supports normal viral replication. During later stages of infection, when enough PB1-F2 protein is synthesized or when cellular mitochondrial apoptotic signaling pathways are activated, the mitochondria undergo a permeability transition.

While induction of apoptosis by influenza virus may at first seem counterintuitive to efficient viral production, it has been shown to be important in influenza viral replication. First of all, influenza virus production is inhibited in stable cell lines overexpressing Bcl-2, while activation of caspase 3 has been shown to be important in replication and propagation of influenza viruses [12–14,42]. In addition, activation of the apoptotic cascade has been suggested to play a role in processing of influenza viral proteins and maturation of viral particles [43]. Moreover, we speculate that sensitization of cells to death by TNFα rather than direct induction of apoptosis by PB1-F2 could have several additional advantageous effects for the virus. TNFα has been shown to exert a strong antiviral effect against influenza virus [44], which is likely to proceed through nuclear factor kappa-B- and c-Jun N-terminal kinase-dependent activation of antiviral gene expression and secretion of antiviral cytokines, such as type I interferon [45]. Sensitization of cells to the proapoptotic effects of TNFα would minimize the antiviral signaling to other cells. In support of this theory, induction of apoptosis by influenza virus has been shown to limit the release of proinflammatory cytokines [46]. Studies are currently underway to analyze a possible differential effect on cytokine induction by the wild-type and PB1-F2 mutant viruses. Finally, as suggested by previous work, immune cells seem to be more sensitive to induction of apoptosis by PB1-F2, as indicated by the finding that *PB1-F2-*knockout influenza virus induced less cell death than the wild-type virus in infected human monocytes. This observation suggests that the protein may play a role in down-regulation of the host immune response to the infection [3]. Interestingly, the ANT3 and VDAC1 proteins are expressed at different levels in a variety of tissues and cell types, including lymphocytes [47–50]. It is thus possible that these proteins may play a role in the regulation of differential apoptotic responses in different cell types following infection with influenza virus. Further animal studies will be needed to evaluate the function of the PB1-F2 protein in modulation of the immune response and its overall role in the pathogenesis of influenza virus infection.

## Materials and Methods

### Cell lines, antibodies, and reagents.

293T, A549, and HeLa cells were obtained from ATCC and were maintained in DMEM culture medium (Gibco, San Diego, California, United States) supplemented with 10% fetal calf serum (Hyclone, South Logan, Utah, United States), 100 U/ml of penicillin G sodium and 100 μg/ml of streptomycin sulfate (Gibco). A549 cells stably overexpressing Bcl-xL were generated by retroviral integration using the pLNCX2 vector system with neomycin selection marker from Clontech (Palo Alto, California, United States). Goat polyclonal antibodies to ANT3 and VDAC1 were obtained from Santa Cruz Biotechnologies (Santa Cruz, California, United States); monoclonal antibodies to Bcl-xL, cytochrome c, Tom20, Tim44 and PARP were obtained from Pharmingen (San Diego, California, United States); rabbit polyclonal antibody to GFP was obtained from Clontech; M30 antibody against cleaved cytokeratin was from Roche (Basel, Switzerland); and antibodies to Flag and HA epitopes were obtained from Sigma (St. Louis, Missouri, United States). Human anti-mitochondrial serum was obtained from Immunovision (Springdale, Arizona, United States). Monoclonal anti-PB1-F2 antibody (clone 26D3) was generated in mice immunized with full-length recombinant PB1-F2 protein produced in *E. coli*. Polyclonal anti-PB1-F2 serum was generated in rabbits immunized with full-length recombinant PB1-F2 protein. BA, cisplatin, and recombinant tBid were obtained from Sigma; G418 and CsA were from Calbiochem (San Diego, California, United States); Mitotracker Red and JC-1 dyes, DAPI, monoclonal anti-COXIV antibody, and secondary fluorochrome-conjugated antibodies were obtained from Molecular Probes (Eugene, Oregon, United States).

### Constructs and cloning.

The pCAGGS vector for the expression of proteins under control of chicken β-actin promoter has been described previously [51]. cDNAs for the full-length ANT3, VDAC1, Bcl-xL, Bak, and Bax were obtained by reverse transcription with oligo-dT primer from RNA obtained from A549 cells. PCR for each gene was performed with gene-specific primers. An N-terminal HA or Flag tag was introduced into each construct by PCR with 5′ gene-specific primers possessing the tag sequences. Each tagged gene was introduced into the pCAGGS vector for mammalian expression and into the pcDNA 3.1^+^ vector (Invitrogen, Carlsbad, California, United States) for in vitro transcription. The Bcl-xL gene was in addition cloned into the pLNCX2 retroviral vector (Clontech) for stable integration into A549 cells. The *PB1-F2* gene was reverse-transcribed and amplified with gene-specific primers by RT-PCR from viral RNA of the influenza virus A/PuertoRico/8/34 strain. N-terminal HA and Flag tags were introduced by PCR as outlined above. Tagged and untagged constructs were cloned into the pCAGGS vector for mammalian expression and the pGEX6P-1 vector (Amersham, Little Chalfont, United Kingdom) for bacterial expression of the GST fusion protein. The pCAGGS-GFP vector was generated by subcloning the GFP gene from the pEGFP vector (Clontech). The HA-tagged PB1-F2-GFP fusion construct was generated by insertion of the full-length HA-tagged *PB1-F2* gene into the pCAGGS-GFP vector. HA-tagged constructs expressing either C- or N-terminally truncated PB1-F2-GFP fusion proteins (HA-nF2-GFP and HA-cF2-GFP, respectively) were generated by insertion of PCR-amplified C- or N-terminal domains into the pCAGGS-GFP vector (see Figure 4). The HA-MTS-nF2-GFP construct was generated by trimolecular ligation of the HA-tagged cytochrome oxidase MTS sequence cloned from A549 cells (sequence available from Clontech) and the N-terminal domain of PB1-F2 using the pCAGGS-GFP vector. Sequences of each generated construct were confirmed by automated sequencing performed at the Mount Sinai sequencing core facility. All primer sequences are available upon request.

### Recombinant protein purification from *E. coli.*

Competent BL-21 cells (Stratagene, La Jolla, California, United States) transformed with pGEX6p-1 vector were cultured to an OD_600_ of 0.6 in 2XYT medium. The cells were induced for 2 h at 37 °C with 1 mM IPTG, collected in PBS, and frozen. Purification of the GST fusion proteins was performed using the GSH Sepharose resin (Amersham) according to the manufacturer's protocol. Purified protein was either eluted with glutathione buffer (Amersham) as a GST fusion protein, or cleaved from GST on the column with Prescission Protease (Pharmacia, Uppsala, Sweden) and eluted with PBS.

### Transfections for localization and apoptosis assays.

A549 and HeLa cells grown on coverslips were transfected in 24-well dishes with 1 μg of vector of interest using Lipofectamine 2000 (Invitrogen) according to the manufacturer's instructions. After 24 h, the cells were fixed with 5% formaldehyde in PBS and permeabilized with 1% Triton X-100. Proteins of interest were visualized by indirect immunofluorescence. Cells were probed with specific primary antibody for 2 h at room temperature, washed, and labeled with secondary antibody conjugated to a specific fluorophore. Labeled cells were visualized by laser scanning confocal microscopy (TCS-SP; Leica, Wetzlar, Germany) with TCS-SP software for image capture. A Zeiss (Oberkochen, Germany) Axiovert 200 microscope with Zeiss Axiovision software was used for fluorescence analysis of live cells. Protocols for assessment of anoikis have been described elsewhere [22]. Briefly, cells were transfected for 24 h, trypsinized, and resuspended in serum-free medium. Cellular morphology was analyzed for blebbing and fragmentation under a phase-contrast microscope over the next hour.

### Mitochondrial purification and cytochrome c release assay.

Freshly isolated Balb/c mouse livers were homogenized and fractionated according to the protocols described previously [52]. Purified mitochondria were resuspended in MRM-S buffer (250 mM sucrose, 10 mM Hepes, 1 mM ATP, 5 mM succinate, 0.08 mM ADP, and 2 mM K_2_HPO_4_ [pH 7.4]) to a final concentration of 10 mg/ml protein. For cytochrome c release assays, 50 μg of mitochondria (by total protein) were incubated with recombinant PB1-F2 or tBid protein for 1 h at 30 °C in a total volume of 25 μl. After incubation, mitochondria were pelleted and the supernatant was collected and analyzed for cytochrome c release by immunoblotting for cytochrome c, while the mitochondrial pellet was analyzed by Western blot for Tom20.

### Determination of the mitochondrial membrane potential with JC-1 fluorescence.

Purified mitochondria (50 μg) were incubated with proteins of interest for indicated times as described above. After incubation, 1 ml of 200 nM JC-1 dye in MRM-S buffer was added to the mitochondria and incubated at RT for 10 min. JC-1 fluorescence was measured in a Bio-Rad (Hercules, California, United States) fluorometer with the excitation filter of 490 nm and emission filter of 590 nm.

### In vivo GST-fusion protein expression and GST pulldowns.

For identification of cellular interactors, 293T cells in 15-cm dishes were transfected with 15 μg of mammalian expression vector encoding GST-fusion proteins of interest. Cells were collected 30 h post-transfection and lysed in coimmunoprecipitation buffer (see below). Lysates were incubated with glutathione beads for 4 h, and beads were spun down and washed ten times in lysis buffer. Proteins were eluted off the beads with glutathione elution buffer, as recommended by the manufacturer's instructions (Amersham).

### Mass spectrometry and protein identification.

The fraction of the eluted proteins was initially separated by SDS-PAGE (10%) and analyzed by silver stain. For mass spectrometry analysis, the eluted proteins were separated on the SDS-PAGE (10%) and visualized by Coomassie blue staining. Bands of interest were cut out from the gel, destained, reduced, alkylated, and digested with trypsin. Micro-HPLC analysis of the tryptic peptides was conducted by using an LCQ electrospray ionization ion trap mass spectrometer (ThermoFinnigan, Waltham, Massachusetts, United States) coupled to an online MicroPro-HPLC system (Eldex Laboratories, Napa, California, United States). Proteins were identified by using the peptide molecular masses and their MS/MS fragment data to search the National Center for Biotechnology Information nonredundant DNA and protein sequence databases with the program KNEXUS (Genomic Solutions, Ann Arbor, Michigan, United States).

### Immunoprecipitations.

For all coimmunoprecipitation experiments, 293T cells in 35-mm dishes were transfected with 1 μg of each appropriate expression vector using Lipofectamine 2000 transfection reagent (Invitrogen) according to the manufacturer's protocol. Cells were collected 30 h post-transfection and lysed in the coimmunoprecipitation buffer: 0.5% NP-40, 150 mM NaCl, 20 mM Hepes (pH 7.4), 1 mM EDTA, 1 mM EGTA, 1 mM DTT, 10% glycerol, and Complete Protease Inhibitor Cocktail and PMSF (Roche). Proteins were incubated at 4 °C overnight with 1 μg of the appropriate antibody, and protein complexes were precipitated with protein G agarose beads (Roche) for 2 h. Beads were washed five times in lysis buffer and resuspended in protein sample buffer. Proteins were subsequently separated by 12% SDS-PAGE and detected by Western blotting.

### In vitro transcription/translation and GST pulldowns.

Proteins were in vitro transcribed and translated by use of a T7-coupled rabbit reticulocyte system (Promega, Madison, Wisconsin, United States) and ^35^S protein labeling mix (Perkin Elmer, Wellesley, California, United States) according to the manufacturers' protocols. For binding experiments, ^35^S-labeled proteins were incubated with 5 μg of either GST-PB1-F2 or GST with glutathione-Sepharose beads for 2 h at 4 °C in binding buffer (PBS with 0.25% NP-40 and 0.1% BSA) and washed with binding buffer three times. The beads were then resuspended in protein sample buffer, separated by 12% SDS-PAGE, and analyzed by fluorography for bound proteins. To visualize ^35^S-labeled proteins by fluorography, the gels were fixed, incubated in Amplify (Amersham), and dried before exposure to film.

## Abbreviations

ANT3: adenine nucleotide translocator 3
BA: bongkrekic acid
CsA: cyclosporine A
GFP: green fluorescent protein
GST: glutathione-S-transferase
HA: hemagglutinin
NP: nucleoprotein
PARP: poly A ribose polymerase
PTPC: permeability transition pore complex
TNFα: tumor necrosis factor alpha
VDAC1: voltage-dependent anion channel 1
